# Ultralow-Noise Chopper Amplifier for Seafloor E-Field Measurement

**DOI:** 10.3390/s24061920

**Published:** 2024-03-17

**Authors:** Sixuan Song, Kai Chen

**Affiliations:** 1 School of Geophysics and Information Technology, China University of Geosciences, Beijing 100083, China; songsx@email.cugb.edu.cn; 2 Key Laboratory of Intraplate Volcanoes and Earthquakes, Ministry of Education, Beijing 100083, China

**Keywords:** chopper amplifier, low noise, electromagnetic receiver, seafloor E-field

## Abstract

The seafloor E-field signal is extremely weak and difficult to measured, even with a high signal-to-noise ratio. The preamplifier for electrodes is a key technology for ocean-bottom electromagnetic receivers. In this study, a chopper amplifier was proposed and developed to measure the seafloor E-field signal in the nanovolt to millivolt range at significantly low frequencies. It included a modulator, transformer, AC amplifier, high-impedance (hi-Z) module, demodulator, low-pass filter, and chopper clock generator. The injected charge in complementary metal-oxide semiconductor (CMOS) switches that form the modulator is the main source of 1/*f* noise. Combined with the principles of peak filtering and dead bands, a hi-Z module was designed to effectively reduce low-frequency noise. The chopper amplifier achieved an ultralow voltage noise of 0.6 nV/rt (Hz) at 1 Hz and 1.2 nV/rt (Hz) at 0.001 Hz. The corner frequency was less than 100 mHz, and there were few 1/*f* noises in the effective observation frequency band used for detecting electric fields. Each component is described with relevant tradeoffs that realize low noise in the low-frequency range. The amplifier was compact, measuring Ø 68 mm × H 12 mm, and had a low power consumption of approximately 23 mW (two channels). The fixed gain was 1500 with an input voltage range of 2.7 mV_PP_. The chopper amplifiers demonstrated stable performance in offshore geophysical prospecting applications.

## 1. Introduction

Natural electromagnetic signals are attenuated by conductive seawater. The high conductivity of dense seawater has long been thought to cause the amplitude of the E-field signal to become weak and difficult to measure. Since the late 1970s, the need for the successful application of seafloor E-field measurements in geophysical exploration [1] has been increasingly recognized by geophysicists. Currently, seafloor E-field measurements are widely used in offshore oil and gas exploration [2], gas hydrate surveys [3], ocean ridge expansion campaign surveys [4], the underwater detection of unknown objects [5], and vessel E-field underwater surveillance for military purposes.

Seafloor oil, gas, and hydrate exploration using the marine controlled source electromagnetic method (CSEM) is required for the transmission of seabed emission currents with a certain frequency excitation signal [6]. The signal transmitted to the seabed below the different media produces an emission wave that arrives at the receiver on the seafloor. The E-field signal is synchronized and recorded using an array of receivers that include artificial and natural source signals. The electrical structure of the seafloor is deduced by analyzing the electromagnetic (EM) field signals recorded by the receiver [7]. The artificial excitation signal frequency band is 0.01 to 100 Hz, and the current amplitude is generally located at 100 to 1000 A. When the transmitter–receiver offset is large, it is difficult for the receiver to measure a weak signal. The amplitude of the natural field source magnetotelluric (MT) signal is weak. This is determined by the signal frequency, water depth, and subsurface electrical structure. The effective observation frequency band for detecting electric fields on the seafloor is approximately 0.0001 to 0.1 Hz [6]. A significant decrease in electric field power occurs on the seafloor at frequencies greater than 0.1 Hz in 1000 m deep water. The natural marine electric field signal is similar to the noise floor of the electric fields (0.1 nV/m/rt (Hz)) at approximately 1 Hz in typical continental shelf environments [6]. Consequently, the receiver should have low noise below 1 nV/rt (Hz) at 1 Hz. To achieve an ocean-bottom E-field receiver with low self-noise, in addition to addressing the need for a low-noise marine E-field sensor, a low-noise post-amplifier should be developed. In MT and CSEM, the instruments are typically used to conduct long-term observations. The high power consumption of the instrument results in more batteries. This requires higher buoyancy to support the added battery weight, which leads to higher costs. Therefore, the post-amplifier should combine low power consumption with compact performance. Additionally, it should have a differential input.

In the widely used low-noise integrated operational amplifiers in the current market, such as Linear LT1028 [8], OPA211 from Texas Instruments [9], and AD797 from Analog Devices, Inc. [10], the voltage noise level is approximately 1 nV/rt (Hz) at 1000 Hz. However, if the 1/*f* noise is significant, these devices cannot meet seafloor E-field measurements with low-noise requirements.

Several integrated circuit chips have been designed as chopper amplifiers for differential inputs and high dynamic ranges. In addition, an auto-zeroing technique in the DC range to *n* × 10^3^ Hz has been applied to achieve an integrated low-noise amplifier. Despite the restrained 1/*f* noise of the integrated chopper and auto-zeroing amplifiers, the background noise remains large. For example, in ADA4528-1 from Analog Devices, Inc. [11] the noise level is approximately 5.6 nV/rt (Hz) at 1 Hz, and the Cirrus Logic CS3301 has a noise level of approximately 8.5 nV/rt (Hz) at 1 Hz [12].

The ultralow-noise amplifier module EM A10 from EM Electronics, Inc. [13] is suitable for use for sensitive measurements, data collection, and systems. The EM A10 was designed with a high gain to reduce the input voltage noise. Its noise level reaches a relatively low level of 0.58 nV/rt (Hz) at 1 Hz without 1/*f* noise, and its input range is ±2 mV when its gain is 2000. Its deficiencies include a limited input range, single-ended input, and size. In terms of certain nanovoltage meters [14], although good noise performance is obtained, they should be operated below a 1 Hz sample rate. Drung and Storm [15] presented an ultralow-noise amplifier with a low-input charge injection of 0.73 nV/rt (Hz) down to a few mHz. Given the single-ended input, power consumption, and input voltage range, it does not satisfy the seafloor E-field application requirements.

In [1], the chopper amplifier designed for an ocean-bottom EM receiver consisted of a modulator, transformer, AC amplifier, demodulator, and DC amplifier. Although it has a low noise level, its high gain (120 dB) reduces the dynamic range. Constable [16] introduced an amplifier device for seafloor E-field signal observations. The amplifier uses chopper amplifier technology that originated in [1]. Following several improvements, the current typical noise was 2 nV/rt (Hz) at 1 Hz, and the power supply range was ±5 V to ±7 V. Compared with the amplifier of [1], its dynamic range, low-frequency responses, and gain (80 dB) were improved. Lower gain means it is harder to achieve equally noise. In [16], a first-order AC coupled circuit was designed and the −3 dB bandwidth of the amplifier was only 0.1 to 20 Hz. Therefore, the low-frequency signal was suppressed, and the signal-to-noise ratio (SNR) was reduced. Ocean-bottom E-field receivers are expected to achieve further improvements in noise power spectral density (PSD), bandwidth, the input range, and power consumption. Moreover, it is crucial to minimize low-frequency noise, specifically 1/*f* noise. This can effectively improve the quality of E-field data.

Based on the aforementioned circumstances, further development is required to reduce 1/*f* noise, lower power consumption, and decrease volume. An ultralow-noise amplifier should be developed to increase the accuracy of marine electric field measurements and offshore field operation time. Based on the conventional method, a high-impedance (hi-Z) module was added to effectively suppress the effect of injected charges and further improve the SNR.

## 2. Principles

**Figure 1:**
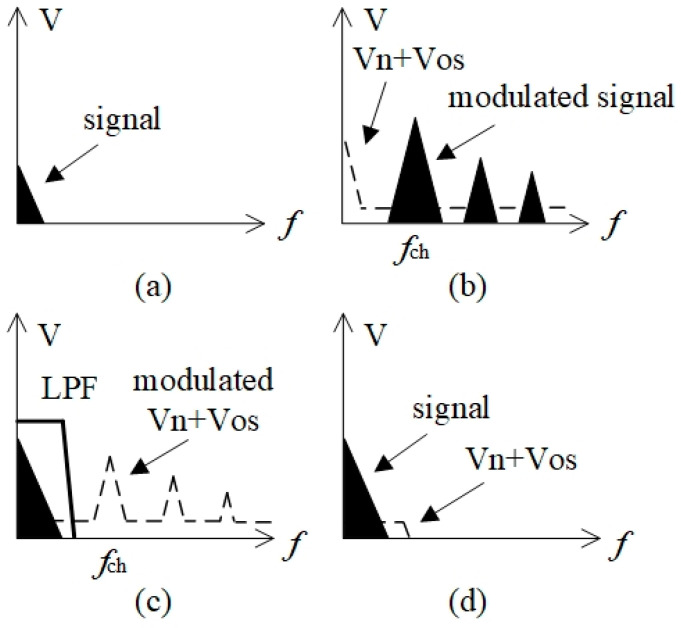
Principle of chopper amplifier. *f*_ch_ is the chopping frequency. (**a**) The original signal; (**b**) the modulated signal; (**c**) the demodulated signal; (**d**) the original baseband signal and the residual noise.

Chopping is a technique that continuously employs the modulation and demodulation processes to separate signals from low-frequency noise and offset voltages. The circuit consists of four basic sections: a modulator, AC amplifier, demodulator, and low-pass filter. Figure 1 illustrates the principle of the chopper amplifier [17]. In the modulator, the original signal is first modulated to a high frequency using a square-wave signal. The modulated signal is then amplified using an AC amplifier. The AC amplifier generates a 1/*f* noise *V*_n_ and DC offset *V*_os_. The 1/*f* noise of the amplifier is high, whereas the high-frequency noise is relatively low. The amplifier amplifies the signal at high frequencies. Thus, the SNR can be improved. The spectra of *V*_n_ and *V*_os_ are shifted to higher frequencies using a synchronous demodulator. The low-pass filter eliminates the effects of *V*_n_ and *V*_os_.

The thermal noise of the transformer secondary resistance, voltage noise, and current noise of the AC amplifier are equivalent to the transformer primary noise, whose input voltage noise of the chopper amplifier is as follows [18]:

(1) $$e_{nc}=\gamma \cdot \sqrt{4KTR+{\left(e_{n}/N\right)}^{2}+{\left(i_{n}N\right)}^{2}\cdot R^{2}}\cdot \sqrt{1+0.8525\frac{f_{k}}{f_{ch}},}$$

where *K* is the Boltzmann constant; *T* is the absolute temperature; *R* is the equivalent input resistance, calculated as shown in formula (2); *N* is the ratio of the secondary to primary turns of the transformer; *e*_n_ is the voltage noise of the AC amplifier; and *i*_n_ is the current noise of the AC amplifier. The abovementioned switch is used to realize the front-end modulator. The transformer is an audio transformer, which is used for impedance matching. Noise figure γ is a constant that depends on the cut-off frequency of the AC amplifier.

(2) $$R=2R_{\mathrm{on}}+R_{\mathrm{pt}}+\frac{R_{\mathrm{st}}}{N^{2}},$$

where *R*_on_ is the on-resistance of the switch, *R*_pt_ is the primary resistance of the transformer, and *R*_st_ is the secondary resistance of the transformer.

According to Fourier expansion, the modulated square wave $M_{1}(t)$ and demodulated square wave $M_{2}(t)$ can be calculated as follows [19]:

(3) $$\begin{array}{cc} M_{1}(t) & =\frac{4}{\pi }[\sin(2\pi f_{\mathrm{ch}}t)+\frac{1}{3}\sin(3\cdot 2\pi f_{\mathrm{ch}}t)+\frac{1}{5}\sin(5\cdot 2\pi f_{\mathrm{ch}}t)+\ldots ],\\ & =\frac{4}{\pi }\sum_{k=0}^{\infty }\frac{1}{(2k+1)}\sin\left[2\pi f_{\mathrm{ch}}(2k+1)t\right], \end{array}$$

(4) $$\begin{array}{cc} M_{2}(t) & =\frac{4}{\pi }[\sin(2\pi f_{\mathrm{ch}}t)+\frac{1}{3}\sin(3\cdot 2\pi f_{\mathrm{ch}}t)+\frac{1}{5}\sin(5\cdot 2\pi f_{\mathrm{ch}}t)+\ldots ],\\ & =\frac{4}{\pi }\;\sum_{l=0}^{\infty }\frac{1}{\left(2l+1\right)}\sin\left[2\pi f_{\mathrm{ch}}(2l+1)t\right]. \end{array}$$

Following synchronous demodulation, the expression is as follows:

(5) $$\begin{array}{c} \begin{array}{cc} V_{dm}\left(t\right) & =AV_{in}\left(t\right)M_{1}\left(t\right)M_{2}\left(t\right),\\ & =AV_{in}\left(t\right)\frac{8}{\pi^{2}}\sum_{k=0}^{\infty }\sum_{l=0}^{\infty }\frac{1}{(2k+1)(2l+1)}, \end{array}\\ \left\{\cos[2\pi f_{ch}(2k+2l+2)t]+\cos[2\pi f_{\mathrm{ch}}(2k-2l)t]\right\}. \end{array}$$

When *k* = *l* = 0, 1, 2…, $V_{dm}(t)$ can be calculated as

(6) $$V_{dm}\left(t\right)=AV_{in}\left(t\right)\frac{8}{\pi^{2}}\sum_{k=0}^{\infty }\frac{1}{{\left(2k+1\right)}^{2}}.$$

When the cut-off frequency is twice the modulation frequency, the signal amplitude is $\frac{4}{\pi }$*A*_max_*V*_in_. The amplitude is $\frac{8}{\pi^{2}}$*A*_max_*V*_in_ after demodulation, resulting in approximately 20% gain loss. Similarly, when the cut-off frequency is four and six times the modulation frequency, the gain losses are approximately 11% and 8%, respectively. If the cut-off frequency is extremely high, the residual offset will increase. Four times the modulation frequency was selected as the cut-off frequency in exchange for balancing the voltage noise and residual offset.

The chopper amplifier modulator was realized using complementary metal-oxide semiconductor (CMOS) switches. The CMOS switches generate injected charges when turned ON or OFF. The injected charges form voltage spikes in the load. Following the application of the demodulator and low-pass filter, the voltage spikes were converted into a residual offset. The residual offset is expressed in the following form [20]:

*V*_os_ = 2τ*V*_spike_*f*_ch,_ (7)

where τ and *V*_spike_ are the time constant and peak of the voltage spike, respectively.

The time constant τ of the voltage spike is generally considerably smaller than that of the half-chopper period. The magnitude of the residual offset is positively related to the modulation clock frequency. Reducing chopper frequency is beneficial for reducing the residual offset. However, the minimum chopper frequency should not be lower than the corner frequency of 1/*f* noise. Therefore, suppressing the injected charge reduces the offset voltage, 1/*f* noise, and corner frequency.

Conventional methods for suppressing injected charges include nested chopping, spike filtering, and dead bands [21,22]. Combined with the principles of peak filtering and dead bands, a hi-Z module was added after the amplifier. At each rising and falling edge of the modulating clock, the output was forced to zero. The demodulator inputs were set to the hi-Z state to reduce the effect of modulator switching noise. Voltage spikes introduced by the injected charge were suppressed in the time domain. Subsequently, the bandpass filter eliminated the effect of voltage spikes in the frequency domain. The tiny time gap set to the hi-Z state was τ_d_. If τ_d_ is larger than the time constant τ of the voltage spike, the offset voltage is effectively suppressed. However, when the demodulation switches are set to the hi-Z state, the output signal is no longer continuous. Therefore, too large a time gap τ_d_ will result in a loss in the total gain.

## 3. Amplifier Design

**Figure 2:**
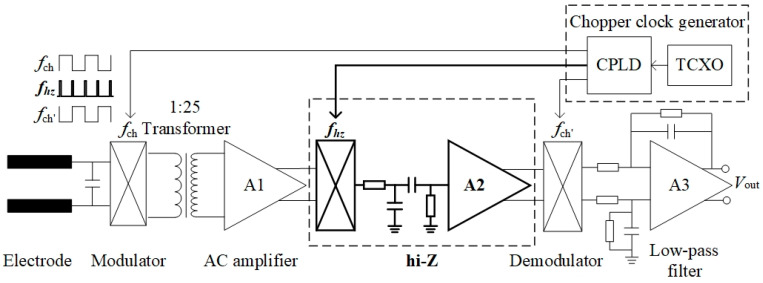
Basic circuit diagram of the chopper amplifier (one channel) (details are omitted for simplicity).

The proposed amplifier contained two channels for the horizontal components Ex and Ey of the E-field signal amplification. The two-channel circuit principle is consistent. Figure 2 illustrates the basic diagram of the chopper amplifier (one channel). The single-channel amplifier comprised a modulator, transformer, AC amplifier, high-impedance (hi-Z) module, demodulator, low-pass filter, and chopper clock generator. In the schematic, the important device parameters are labeled accordingly.

The front-end modulator was realized using high-speed CMOS switches with a significantly low on-resistance of approximately 1.5 Ω. It was controlled using a chopper clock *f*_ch_. The signal is connected to an audio transformer with a transformer ratio of 1:25. The chopper amplifier gain can be increased by improving the transformer turn ratio, for example, by reducing the number of input coil turns, increasing the number of output coil turns, or increasing the gain of the internal AC amplifier. In terms of the transformer, using extremely few input coil turns reduced the input impedance of the chopper amplifier. Conversely, having a large number of output coil turns increased the output impedance, causing the current noise of the AC amplifier to become a main source of noise, leading to an increase in the total output noise. The differential output of the transformer was connected to the input of an AC-coupled amplifier. The INA821 amplifier (Texas Instruments, America) was used to build an AC-amplifying circuit that involves a low-power instrumentation amplifier. The INA821 current noise was 130 fA/rt (Hz) at 1 kHz and the consumption current was 650 μA (maximum). Low noise and a high input range can be achieved if the characteristics of the transformer output coil match those of the best source impedance of the integrated operational amplifier. To obtain the best ratio, the current and voltage noises of the AC amplifier should be considered. Using the INA821 to build an AC amplifier, the noise floor was approximately 7 nV/rt (Hz) at 1 kHz. Instead of a conventional transistor amplifier, an integrated operational amplifier was used for the AC amplifier, which is the main noise contributor, to reduce its noise contribution for a small amount of degradation in power dissipation. The −3 dB bandwidth remained at 20 Hz to 20 kHz, and the flat pass-band gain was set to 40.

Generally, an AC amplifier’s output square wave is synchronously rectified by using a demodulator. The hi-Z module is added between the two stages. To balance the relationship between 1/*f* noise and total gain, this module is set into a hi-Z state for 300 ns after each clock edge. The bandpass filter included in the module further reduces the effect of the switching spikes in the front-end modulator. A CMOS device (ADG613, Analog Devices, America) containing four independent single-pole, single-throw (SPST) switches is used to realize a synchronous demodulator. The demodulator is designed based on the principle of coherent detection. Two switches are turned on with a logic low on the appropriate control input, with the other two switches having inverted logic. Only one clock is required to demodulate the differential signal to ensure complete synchronization. These switches offer ultralow charge injection of 1 pC over the input signal, and the on-resistances are 85 Ω. An auto-zeroing amplifier (OPA387, Texas Instruments, America) with no obvious 1/*f* noise is used as the low-pass filter. Therefore, it does not introduce an additional 1/*f* noise. The low-pass filter has a cut-off frequency of 300 Hz. The voltage follower, which uses the same auto-zeroing amplifier, increases the output impedance. The chopper clock generator includes a complex programmable logic device (CPLD) and a crystal oscillator. The CPLD outputs three square waves. In this system, the modulator and demodulator are sync-driven by a 2 kHz sync clock. With 0.6 mW of power consumption at 12 MHz output frequency, the SiT8021 µPower oscillator (SiTime, America) is ideal for power-sensitive applications. The crystal oscillator frequency is equal to or higher than 12 MHz to provide a tiny time gap for the hi-Z module.

The chopper amplifier has a theoretical open-loop low gain of 2000. Based on the AC amplifier gain multiplied by that of the transformer, the difference in the single-ended region contributes to the double gain. Considering the efficiency of the synchronous demodulator, the fixed gain of the chopper amplifier can reach 1500. The chopper amplifier gain can be increased by increasing the internal AC amplifier gain. A lower chopper amplifier gain results in a larger input range of useful signals, although the equivalent input noise increases. Meanwhile, the input range decreases as the gain increases and the dynamic range decreases. Following a series of experiments with different amplifier gains, we found the best gain range to be approximately 1500 for a balance between the input range and equivalent input noise. 

The supply voltage was ±2.5 V, while the quiescent current was approximately 1.6 mA per channel. A signal generator (DG1022, Rigol, China) outputted a sine signal with a frequency of 1 Hz and amplitude of 2 mV_pp_. The signal amplitude was gradually increased in steps of 0.1 mV_pp_. An oscilloscope (MSO7014B, Agilent, America) was used to observe the output of the chopper amplifier. When the input was 2.7 mV_pp_, the output waveform was distorted. The maximum output could achieve 4.05 V_pp_ and be used directly because the single-ended output completely matched the input of the following acquisition module.

**Figure 3:**
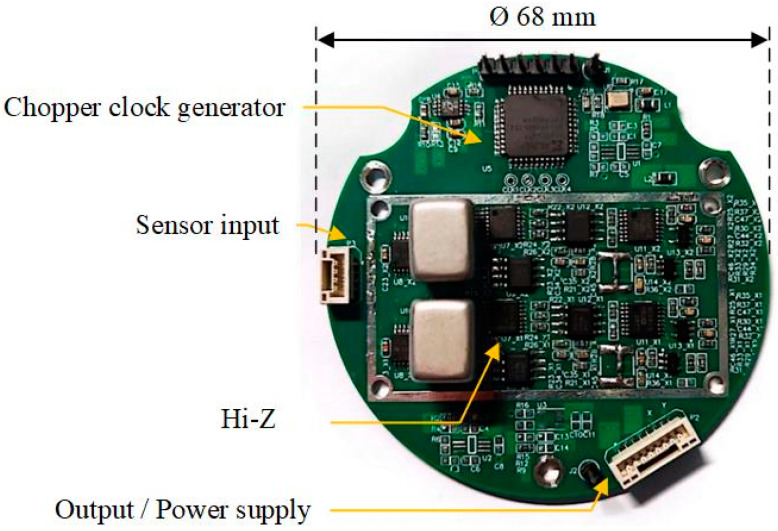
Photograph of the ultralow-noise chopper amplifier with two channels. Left interface: sensor input; bottom right interface: signal output and power supply. The unsoldered part functions as a backup power module, supporting a broad range of power inputs.

Figure 3 illustrates a photograph of the ultralow-noise chopper amplifier. A customized case of suitable size made of annealed mu metal was used to eliminate all the electromagnetic interference that the audio transformers might have had. Moreover, the size of the printed circuit board (PCB) was minimized by selecting suitable components and optimizing their layouts. The PCB had a circular shape with a diameter of only 68 mm.

## 4. Amplifier Performance

To evaluate the amplifier performance comprehensively, we tested parameters including the frequency response, voltage noise, power consumption, input resistance, input voltage range, and maximum non-distortion output voltage.

**Figure 4:**
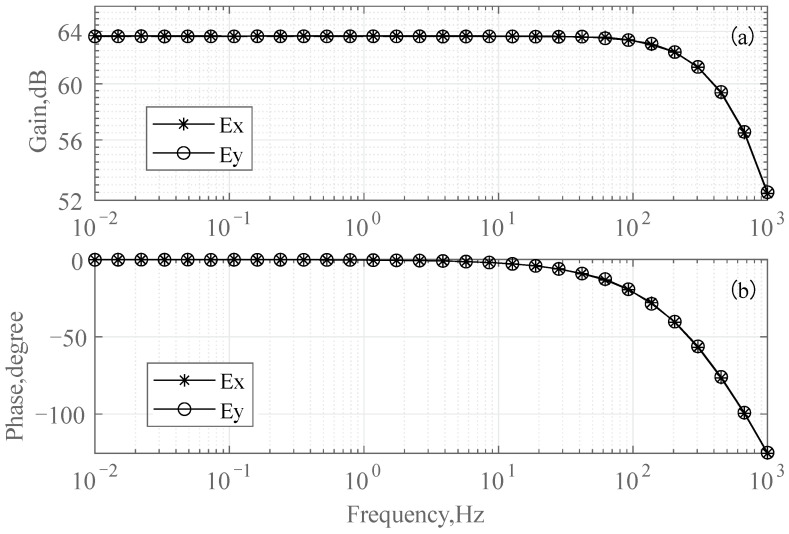
Characteristics of the gain and phase relative to frequency. (**a**) Amplifier magnitude vs. frequency response. (**b**) Amplifier phase vs. frequency response. The low-pass filter −3 dB bandwidth range is 300 Hz. Ex and Ey represent the two channels of the ultralow-noise chopper amplifier, respectively. The frequency responses of the two channels nearly coincide.

Frequency response measurements were performed using a dynamic signal analyzer (35670A, Agilent). Figure 4 illustrates the measured characteristics of the gain and phase of the ultralow-noise chopper amplifier against frequency. For a gain of 64 dB, a −3 dB cut-off frequency of 300 Hz was achieved over the band of interest.

**Figure 5:**
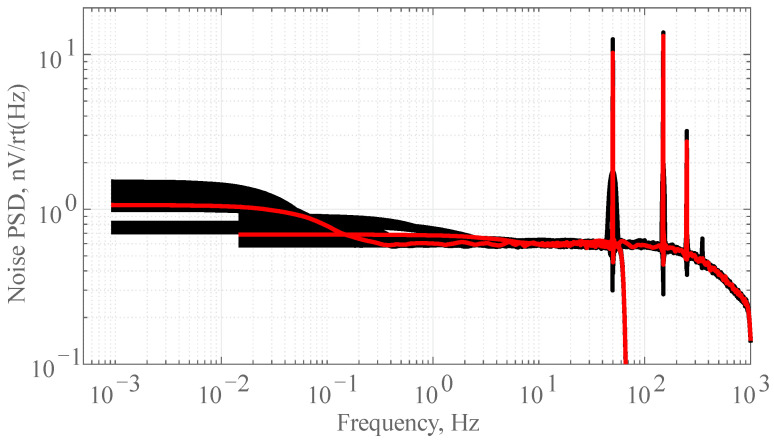
Voltage noise PSD measured characteristics of *e*_n_ against frequency. There are 20 black lines for 20 segments. The red line is the average result. Ultralow voltage noise of 0.6 nV/rt (Hz) at 1 Hz and 1.2 nV/rt (Hz) at 0.001 Hz with a 1/*f* corner below 100 mHz is demonstrated.

For voltage noise measurements, we shortened the input of the chopper amplifier and placed it in a three-layer permalloy shielding box to prevent external electromagnetic interference. A low-noise data logger was used to record the output noise of the chopper amplifier. The sampling rate of the special data logger was set to 150 Hz and 2400 Hz to ensure the accuracy of the low-frequency noise. The typical noise level was 500 nV/rt (Hz) and 200 nV/rt (Hz) at 0.001 Hz and 1 Hz, respectively. Power was supplied via a 25.2 V 2 Ah Li-ion battery. The data logger supports outputting a low-noise ± 2.5 V voltage to power the ultralow-noise chopper amplifier. The data logger and Li-ion battery were placed inside another shielding box. The box was well connected to the ground. For the low-frequency noise PSD measurement, we conducted a 6 h output noise recording, as shown in Figure 5.

To measure the power consumption, a DC power source (E3648A, Agilent) was used to supply the power. The output was set as ±2.5 V. With two channels working together, the positive and negative currents were 6 and 3 mA, respectively. Therefore, the power consumption of the chopper amplifier was 23 mW. The positive and negative current values were different because the chopper clock generator only required positive power supply (+2.5 V, +3 mA). The chopper clock generator and power module consumed a total power of 8 mW.

Based on the aforementioned test results, Table 1 summarizes the voltage noise, −3 dB bandwidth, maximum input range, input resistance, and power consumption.

Table 2 summarizes the implemented chopper amplifier and the recently reported state-of-the-art chopper amplifiers. Compared with the EM A10, this study provides the advantages of a differential input mode, power consumption, and size. As summarized in the table, compared with other studies, our chopper amplifier exhibits lower noise and power consumption. The results demonstrate that the proposed chopper amplifier architecture and design methodology are suitable for a range of DC to 300 Hz or higher frequencies for seafloor E-field measuring applications.

## 5. Application Examples

**Figure 6:**
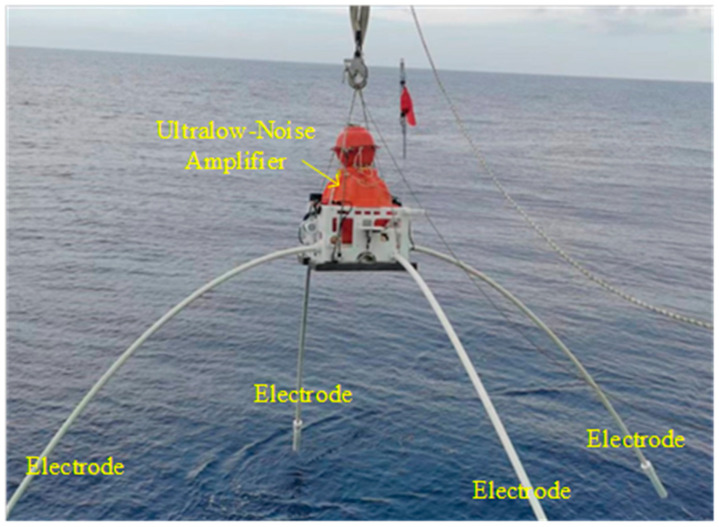
MicrOBEM receiver prepared for deployment. Four electrodes are installed on the receiver. The electric dipole arms have polypropylene pipes terminated with silver–silver chloride electrodes. Dipole cables run along the insides of the electrode arm. The ultralow-noise amplifier is placed in a glass sphere.

**Figure 7:**
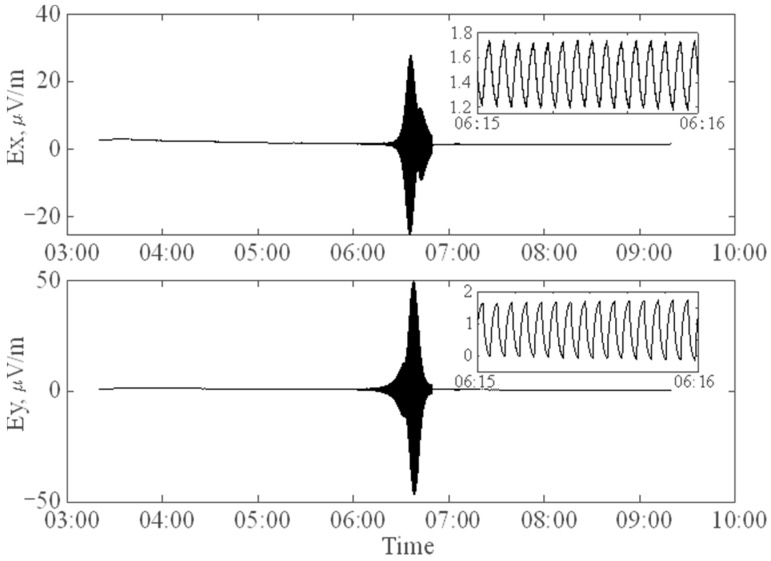
Sample time series collected from transmitted CSEM and natural varied MT signals. The receivers show the 0.25 Hz signal from the transmitter. The Ey recording (**bottom**) is slightly larger than that of Ex (**top**), with both receiver antennas being 8 m. The horizontal axis indicates time, expressed in hh:mm.

Examples of these applications are presented in this section. Field test observations were conducted to evaluate the field performance of the receiver containing the chopper amplifier. Figure 6 illustrates the offshore field deployment of the MicrOBEM receiver. In 2023, an offshore field experiment was performed in the central basin of the South China Sea using the Shi Yan Liu Hao research/survey vessel. The water depth was 5000 m. This experiment aimed to confirm the noise level of the chopper amplifier. The transmission current was 125 A_p_ and the transmission dipole length was 300 m in this CSEM application. Figure 7 shows time series samples from the CSEM and MT signals.

**Figure 8:**
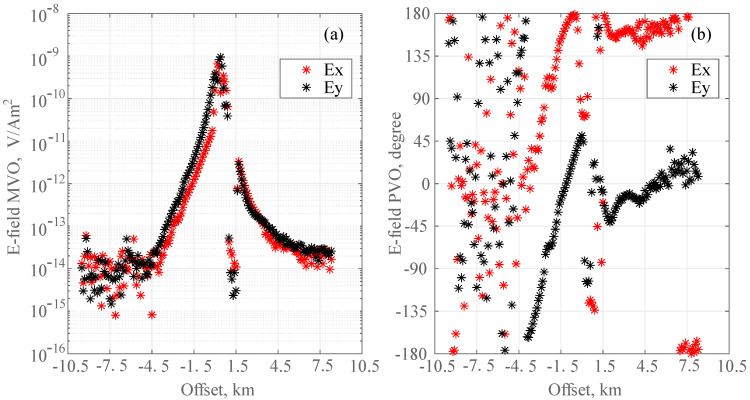
Magnitude and phase relative to source–receiver offset. (**a**) Amplitude. (**b**) Phase vs. offset between the transmitter and receiver for 3 km/h speed and 0.25 Hz transmission tow.

The E-field time series from the receivers was Fourier transformed to the frequency domain within stack frames of 60 s. The results were merged with the navigation source–receiver offset. Figure 8 shows the magnitude and phase versus the source–receiver offset for the receiver. The 0.25 Hz data are above the instrument noise floor at a 4.5 km range. The noise floor for the horizontal E-field was approximately 10^−14^ V/Am^2^. At approximately 1.4 km, the transmitter was out of action for 15 min owing to a shipboard power failure. When the transmitter had no signal, or the signal was below the receiver’s noise floor, the phase data became scattered.

## 6. Conclusions

A chopper amplifier with low noise, low power consumption, and differential input was proposed in this study. It exhibited low noise, particularly in the low-frequency band. In the frequency range less than 100 mHz, there was still no evident 1/*f* noise. Low-frequency noise is ultralow noise of 0.6 nV/rt (Hz) and 1.2 nV/rt (Hz) at 1 Hz and 0.001 Hz, respectively. Post-application and offshore tests were conducted and the results are presented. We demonstrated that low-noise chopper amplifiers are suitable for CSEM applications. The state-of-the-art results of the proposed chopper amplifier indicate that it can effectively serve as a low-noise amplifier in seafloor E-field receiver front ends.

## Figures and Tables

**Table 1:** Chopper Amplifier Parameters.

**Number of Channels**	2
**Voltage noise**	0.6 nV/rt (Hz) @ 1 Hz 1.2 nV/rt (Hz) @ 0.001 Hz
**−3 dB bandwidth**	DC–300 Hz
**Input range**	2.7 mV_pp_ @ 1 Hz
**Gain (flat band)**	64 dB
**Power consumption**	~23 mW
**Supply voltage**	±2.5 V
**Dimension**	Ø 68 mm × H 12 mm

**Table 2:** Summary of implemented chopper amplifier and recently reported state-of-the-art chopper amplifiers.

	*e*_n_ (nV/rt (Hz) @1 Hz)	1/*f* (mHz)	Input Voltage Range (mV_PP_)	Gain (dB)	Supply Current (mA)	Dimension	Supply Voltage (V)	−3 dB Band Width (Hz)	Input Mode
EM A10	0.58	Unclear	4	66	2 (one channel)	L 120 mm × W 70 mm × H 35 mm	±6	DC–10 k	Single-ended
Drung and Storm [15]	0.73	3	6	60	65 (one channel)	Unclear	±5	DC–300	Single-ended
Constable [16]	2	Unclear	<1.4	80	Unclear	Unclear	±5–7	0.1–20	Differential
This work	0.6	<100	2.7	64	+6, −3 (two channels)	Ø 68 mm × H 12 mm	±2.5	DC–300	Differential

## Data Availability

The datasets generated and/or analyzed during the current study are available from the corresponding author on reasonable request.
